# Supplementary material for: The interactome of intact mitochondria by cross-linking mass spectrometry provides evidence for coexisting respiratory supercomplexes
Source: Mol Cell Proteomics. 2017 Dec 8;17(2):216–32. doi: 10.1074/mcp.RA117.000470 (PMC5795388; doi:10.1074/mcp.RA117.000470)
Supplement: Supplemental Data [file supp_RA117.000470_133922_0_supp_23978_fzffwf.zip › spectra_annotation/mito_spectra_annotation/364-1-1-1-4-1.pdf]

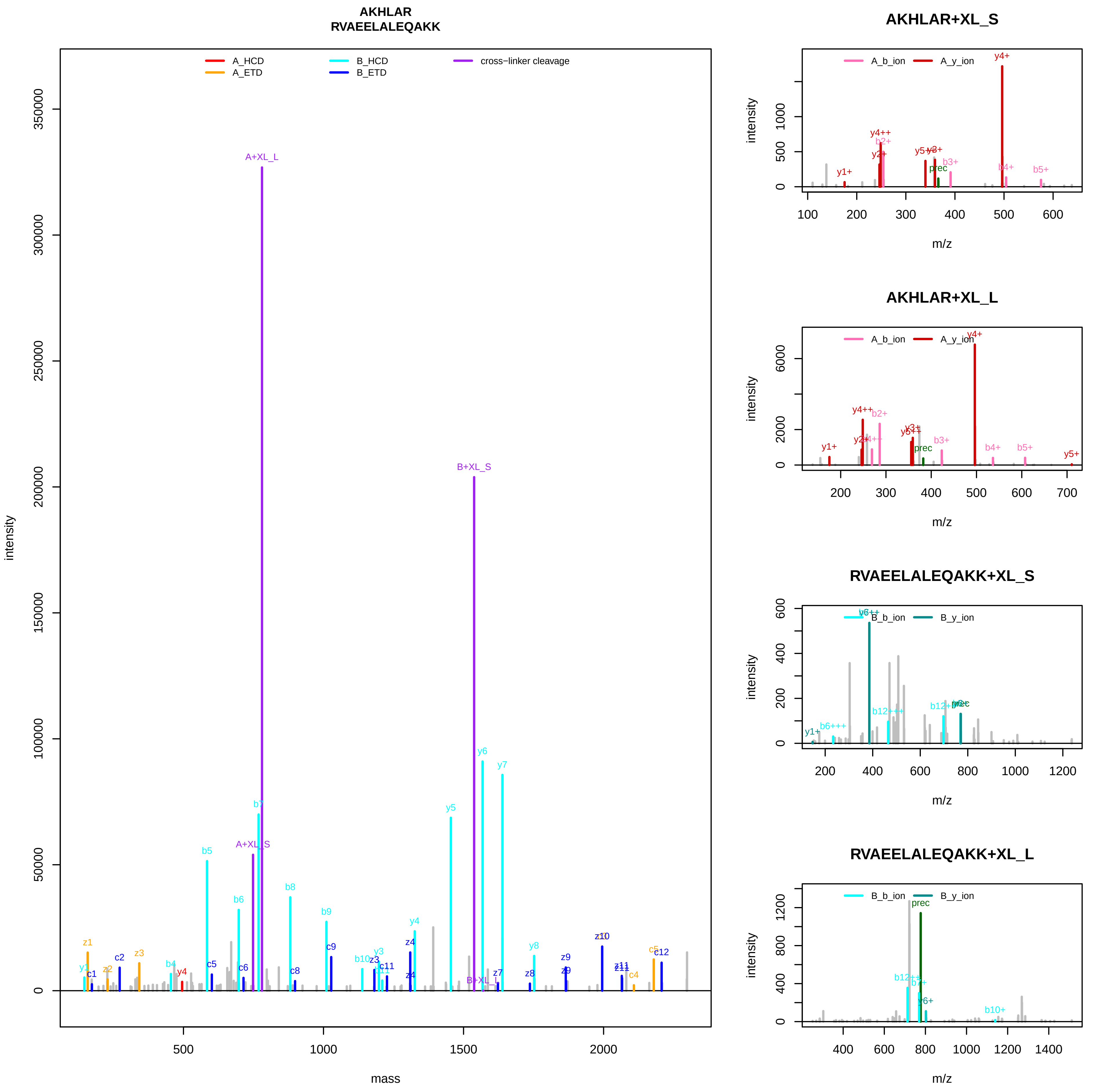
AKHLAR
RVAEELALEQAKK
A_HCD
A_ETD
B_HCD
B_ETD
cross−linker cleavage
A+XL_L
B+XL_S
A+XL_S
B+XL_L
intensity
mass
y1
c1
z1
z2
c2
z3
b4
y4
b5
c5
b6
c6
b7
b8
c8
b9
c9
b10
z3
y3
c11
z4
y4
y5
y6
y7
z7
z8
y8
z9
z10
z11
c4
c5
c12
AKHLAR+XL_S
A_b_ion
A_y_ion
y1+
y2+
y4++
b2+
y5+
y3+
prec
b3+
y4+
b4+
b5+
m/z
AKHLAR+XL_L
RVAEELALEQAKK+XL_S
B_b_ion
B_y_ion
y1+
b6+++
y6++
b12+++
b12++
y6+
RVAEELALEQAKK+XL_L
b12++
b7+
y6+
b10+
